# Drug Target Mining and Analysis of the Chinese Tree Shrew for Pharmacological Testing

**DOI:** 10.1371/journal.pone.0104191

**Published:** 2014-08-08

**Authors:** Feng Zhao, Xiaolong Guo, Yanjie Wang, Jie Liu, Wen-hui Lee, Yun Zhang

**Affiliations:** 1 Key Laboratory of Animal Models and Human Disease Mechanisms of the Chinese Academy of Sciences & Yunnan Province, Kunming Institute of Zoology, Chinese Academy of Sciences, Kunming, Yunnan, PR China; 2 Kunming College of Life Science, University of Chinese Academy of Sciences, Kunming, Yunnan, PR China; University of Bologna & Italian Institute of Technology, Italy

## Abstract

The discovery of new drugs requires the development of improved animal models for drug testing. The Chinese tree shrew is considered to be a realistic candidate model. To assess the potential of the Chinese tree shrew for pharmacological testing, we performed drug target prediction and analysis on genomic and transcriptomic scales. Using our pipeline, 3,482 proteins were predicted to be drug targets. Of these predicted targets, 446 and 1,049 proteins with the highest rank and total scores, respectively, included homologs of targets for cancer chemotherapy, depression, age-related decline and cardiovascular disease. Based on comparative analyses, more than half of drug target proteins identified from the tree shrew genome were shown to be higher similarity to human targets than in the mouse. Target validation also demonstrated that the constitutive expression of the proteinase-activated receptors of tree shrew platelets is similar to that of human platelets but differs from that of mouse platelets. We developed an effective pipeline and search strategy for drug target prediction and the evaluation of model-based target identification for drug testing. This work provides useful information for future studies of the Chinese tree shrew as a source of novel targets for drug discovery research.

## Introduction

The Chinese tree shrew (*Tupaia belangeri chinensis*), which is widely distributed throughout Southeast Asia and Southwest China [1], possesses many unique characteristics that contribute to its suitability as an experimental animal. Our previous phylogenomic analyses revealed that this animal is closely related to primates, with both shared and unique features useful for characterizing nervous and immune system signaling pathways[2]. Until now, Many attempts have been made to use the tree shrew as an animal model for hepatitis C virus infection [3], hepatitis B virus infection [4], [5], myopia [6], social stress and depression [7] and bacterial infection [8]. Despite recent progress, various obstacles continue to hinder the establishment of the tree shrew as an animal model, including a lack of detailed knowledge, such as its genetic background, drug targets.

However, because of the high quality of the tree shrew genome and the need for better animal models for *in vivo* pharmacological testing, we nevertheless believe that the tree shrew is a promising model for drug target discovery. Drug targets and their products is the core elements required to develop better animal models for drug discovery. The interaction of these targets with ligands can modulate the function of many classes of pharmaceutically useful proteins, including enzymes, G-protein-coupled receptors (GPCRs), ion channel proteins and nuclear receptors [9], [10]. Many computational approaches have been developed to analyze and predict drug targets based on compound-protein interactions [11], [12]. However, these studies primarily focused on searches for new drugs or targets, and approaches appropriate for high-throughput genomic or transcriptomic screening of drug targets have not been developed. In this present study, we addressed the important problem of drug target identification in drug discovery. Then, we developed a useful pipeline and stringent search strategy for large-scale genomic and transcriptomic screening of candidate proteins. In addition, we optimized this strategy for drug target identification.

## Results

### Transcriptome assembly and annotation

**Figure 1:**
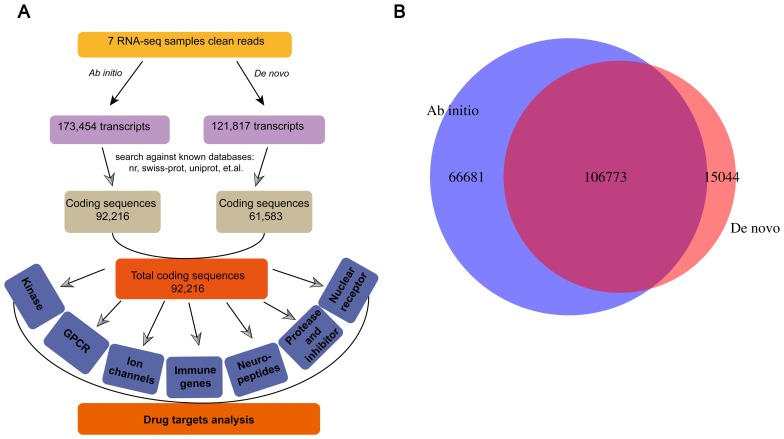
Overview of Chinese tree shrew data assembly for drug target analysis. (A) The pipeline of assembled data for drug target analysis. (B) A Venn diagram illustrating the overlap of transcripts obtained from the *ab initio* and *de novo* assembly methods. Most transcripts obtained from the two different assembly methods shared the same complete sequence or coding sequence.

We developed a pipeline for the global annotation of transcripts from RNA sequencing (RNA-seq) data. Because we focused on drug target proteins or domains, our information retrieval system was biased toward predicted protein-coding transcripts (such as homologs of known genes or those with coding potential). Transcriptome assembly was performed using both *ab initio* and *de novo* methods. Using the *ab initio* assembly programs Tophat and Cufflinks, 173,454 transcripts were obtained from seven different tree shrew tissues (brain, heart, liver, kidney, pancreas, ovary and testis). Transcriptome *de novo* assembly was performed using the SOAPdenovo-trans program to identify 121,817 transcripts (Figure 1A). To maximize the detection of protein-coding transcripts, we used a less stringent but broadly adopted definition that considers a sequence to be protein-coding if it contains the complete coding DNA sequence (CDS) [13]. As a result, 53.2% of the 173,454 transcripts assembled *ab initio* from 107,429 loci included regions annotated as CDSs. A total of 50.6% of the 121,817 transcripts assembled *de novo* were annotated as CDSs and aligned with 100% identity to the transcripts assembled by Cufflinks. A comparison of the transcriptomes generated by the two assembly methods (Figure 1B) revealed that 106,773 transcripts could be assembled using either method, although thousands of novel transcripts were identified separately. Consequently, subsequent analyses were based on protein-coding transcripts obtained via *ab initio* assembly.

### Candidate drug target proteins

To identify potential drug targets, we surveyed the tree shrew transcriptome for the following common targets of existing pharmaceuticals: kinases, GPCRs, ion channel proteins and nuclear receptors. Three classes of immune-related proteins, as well as neuropeptides, proteases and inhibitors, were also predicted as candidate drug targets. As described below, 9,756 transcripts were identified as candidate drug targets for further analysis.

**Figure 2:**
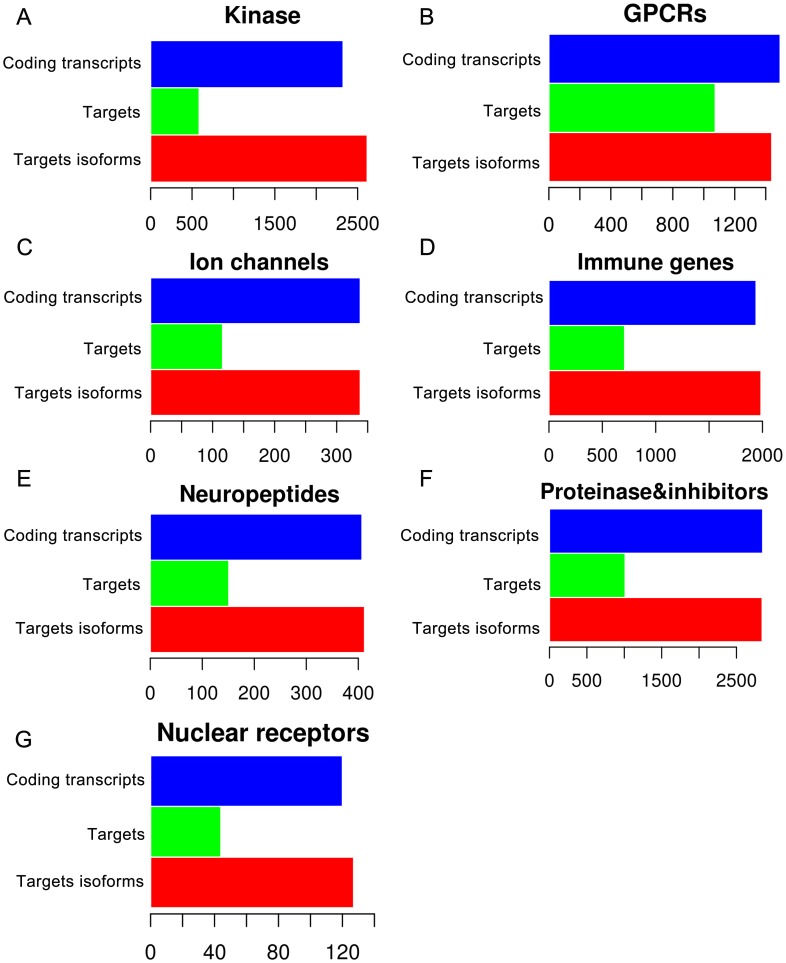
Proteins in the tree shrew identified as potential drug targets. (A) 586 kinase proteins with 2,324 gene isoforms were predicted as drug targets from 2,614 coding transcripts. (B) 1,074 GPCR proteins were predicted from 1,439 coding transcripts divided into four classes. (C) 116 ion channel proteins were predicted from 337 coding transcripts. (D) 709 immune genes were predicted from 1,986 coding transcripts. (E) 151 neuropeptides were predicted from 412 coding transcripts. (F) 1,011 proteinases and inhibitors were predicted from 2,842 coding transcripts. (G) 44 nuclear receptors were predicted from 126 coding transcripts.

Based on Pfam domain annotation, 2,614 coding transcripts with kinase domains were extracted from the tree shrew transcriptome gene sets (Figure 2A; Table S1 in File S1). OrthoMCL was used to cluster these domains into 129 groups based on a reference dataset (KINBASE) combined with phylogenetic analysis. Tree shrew GPCRs were identified using HMMTOP and GPCRDB searching, resulting in 1,439 tree shrew GPCRs that coded transcripts classified and annotated into four families: the rhodopsin family (*n*  =  1,244), the secretin receptor family (*n*  =  115), the metabotropic glutamate receptor family (*n*  =  47) and others (*n*  =  33) (Figure 2B; Table S2 in File S1). Similar to results from other species, 47.6% of the GPCR transcripts were annotated as olfactory receptors. To search for ion channel proteins, vertebrate ligand-gated ion channel (LGIC) subunits were used in BLAST queries against the tree shrew transcriptome. After ranking the resulting hits based on *E*-values (1e-10 cutoff) and removing redundant sequences, 337 ion channel-coding transcripts were identified (Figure 2C; Table S3 in File S1). To identify genes of the immune system, we performed a database query of the tree shrew transcriptome against the Immunome database. Using a cutoff *E*-value of 1e-10, 1,986 immune-coding protein transcripts were identified in the transcriptome (Figure 2D; Table S4 in File S1). Scanning for neuropeptides was performed using a stringent search against NeuroPedia followed by manual comparison to similar species. Combined with the results of the database query search, we recovered 412 neuropeptide coding transcripts from the tree shrew transcriptome (Figure 2E; Table S5 in File S1). A total of 2,842 coding transcripts of putative peptidases and peptidase inhibitors were detected using the MEROPS database server (Figure 2F; Table S6 in File S1). Similar to numbers reported for other primate species, 120 protease gene families were identified in the tree shrew. Nuclear receptors were detected by KEGG annotation, resulting in the identification of 126 putative nuclear receptor-coding transcripts in the tree shrew (Figure 2G; Table S7 in File S1).

### Drug target analysis

To identify potential drug targets, a stringent search strategy was established in which weights were assigned to rank the entire set of potential drug targets. Criteria used for weighting included associated compounds, expression in different tissues and domain information (Table 1). Using this weighting scheme, 3,482 different proteins (8,782 gene isoforms) were identified as potential drug targets among 9,756 candidate proteins; of these, 446 to 1,049 with the highest possible drug scores or total scores had drug leads (i.e., known drugs or approved compounds) (Table S8 in File S1).

**Table 1:** Criteria for the search strategy used in drug target rankings.

Criteria (default weighting)	Weighting
FPKM^1^ > 10 in each tissue	0.1
Essentiality^2^	0.1
Drug literature: hit to ChEMBL	0.25
Drug literature: druggable in ChEBML	0.75
Drug literature: tractable in ChEBML	0.25
DrugBank: hit to DrugBank approved list	1
No DrugBank approved target, although clinical data are available	0.25
TTD: protein is orthologous to TTD-successful target	1
TTD: No TTD-successful target but matches compound target	0.25
Lead compound found	1
The protein is a GPCR or a protein kinase	1
No lead compound but presence of drug domain	0.2

1 FPKM represents Fragments Per Kilobase of transcript per Million mapped reads.

2 Essentiality, lethal phenotype in mouse ortholog knockout or lethal were considered indicative of essential genes in tree shrew.

**Figure 3:**
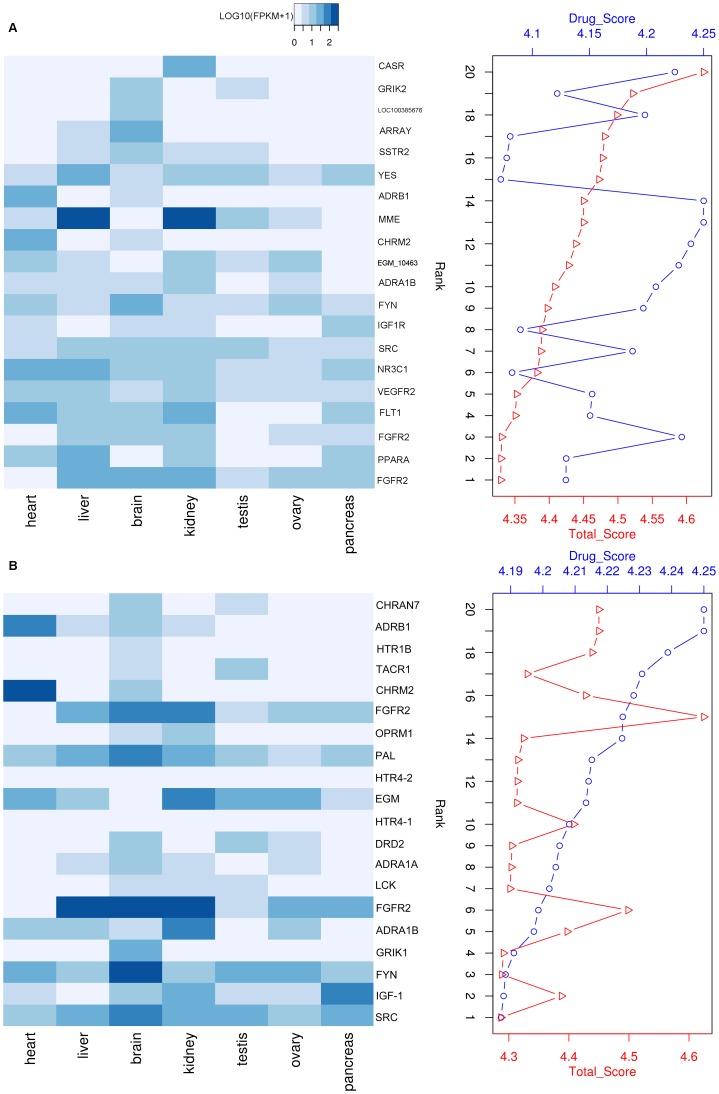
Top 20 drug targets ranked by weighted profiles. (A) Top 20 drug targets ranked by total score. FPKM values of each drug target in seven tissues represent its expression level in each tissue. (B) Top 20 drug targets ranked by drug score. Expression scores were not used to calculate drug scores.

To determine whether total score ranking is an appropriate indicator of candidate drug target quality, we compared drug scores and total scores between the top 20 drug targets. High total scores were associated with drug targets having high expression in tissues, particularly in the liver and kidneys (Figure 3A). Unlike the total scores, expression patterns were considered when calculating the drug score of each drug target. Our comparative analysis revealed that half of the drug targets with high drug scores were expressed at low levels in tissues (Figure 3B). Total scores accurately reflected the quality of all potential drug targets, indicating that target drug expression is an important predictor of drug quality. Based on the total score, the top 20 tree shrew drug targets (Table 2) included several homologs of targets for cancer chemotherapy, including vascular endothelial growth factor receptor 2 (VEGFR2), membrane metallo-endopeptidase (MME), and the proto-oncogene tyrosine-protein kinase Yes (YES1). In addition, as expected, several targets (NR3C1, GSK3B, EGM_09788) with high scores were related to depression and age-related decline. This result is consistent with the report of Fuchs [7].

**Table 2:** Top 20 promising drug targets in *Tupaia belangeri chinensis*.

Gene_id	Annotation	Num_Isoforms	ChEMBL hit	Drug_name	Tissue_expression¶	Drug_Score	Total Score	Rank
XLOC_015532	FGFR2	2	2064724|3CLY	Palifermin, Thalidomide	L,B,K	4.22	4.62	1
XLOC_082803	PPARA	2	2073686|3ET1	Multi-drug (Clofibrate, e.g)	H,L,K	4.12	4.52	2
XLOC_015530	FGFR2	6	149827|2PSQ	Palifermin, Thalidomide	B,K	4.20	4.50	3
XLOC_087227	FLT1	2	2038422|2QU5	Sunitinib	H,L,K	4.08	4.48	4
XLOC_014821	VEGFR2	7	2038422|2QU5	Sorafenib,Sunitinib	H,L,K	4.08	4.48	5
XLOC_017786	NR3C1	4	84768|1M2Z	Multi-drug (Flunisolide, e.g.)	H,L,K	4.07	4.47	6
XLOC_015794	I79_002622	2	145905|1Y57	Dasatinib	B	4.25	4.45	7
XLOC_080516	IGF1R	1	2209608|3NW5	Multi-drug (Insulin recombinant, e.g)	P	4.25	4.45	8
XLOC_055323	FYN	6	2013715|2DQ7	Dasatinib	B	4.24	4.44	9
XLOC_084356	ADRA1B	4	2044786|2VT4	Multi-drug (Midodrine, e.g.)	K	4.23	4.43	10
XLOC_067939	EGM_10463	2	2055182|2ZK2	Multi-drug (Icosapent, e.g.)	K	4.21	4.41	11
XLOC_018597	CHRM2	2	2044786|2VT4	Multi-drug (Succinylcholine, e.g.)	H	4.20	4.40	12
XLOC_033229	MME	3	151205|2QPJ	Candoxatril	L,K,T	4.09	4.39	13
XLOC_076965	ADRB1	1	2044789|2VT4	Multi-drug (Esmolol, e.g.)	H	4.19	4.39	14
XLOC_039195	YES	4	145905|1Y57	Dasatinib	L,K	4.08	4.38	15
XLOC_021221	SSTR2	2	2034098|2PED	Octreotide, Vapreotide	B	4.15	4.35	16
XLOC_051598	ARRAY	3	2100072|3LII	Multi-drug (Choline, e.g.)	B	4.15	4.35	17
XLOC_050569	LOC100385676	3	2085214|3H6H	L-Glutamic Acid, Topiramate		4.23	4.33	18
XLOC_001891	GRIK2	4	118864|1S50	Multi-drug (Topiramate, e.g.)	B	4.13	4.33	19
XLOC_075660	CASR	2	2014833|2E4X	Cinacalcet	K	4.13	4.33	20

¶ FPKM > 10; tissue abbreviations: H, heart, L, liver, B, brain, K, kidney, T, testis, O, ovary, P, pancreas.

**Figure 4:**
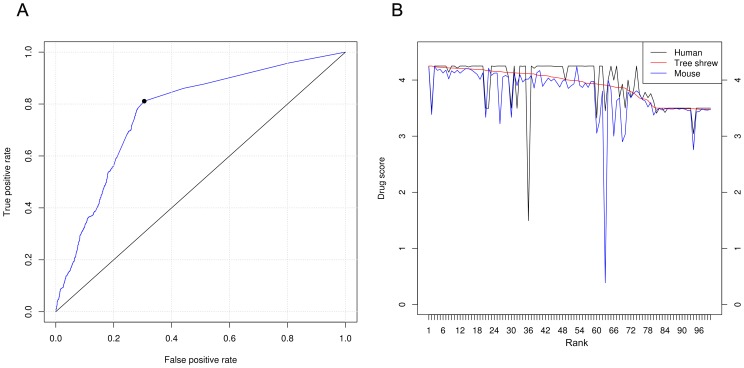
Performance of drug targets in the tree shrew. (A) ROC curves for predicted drug targets indicating high performance. (B) Comparison of tree shrew drug scores to those of human and mouse orthologs. The plot depicts the top 100 tree shrew drug scores.

To evaluate our predictions, the predictor performance was measured using a receiver operating curve (ROC). Drug Bank targets were treated as the reference standard. As shown in Figure 4A, the AUC (area under the ROC curve) was attained for more than 80% of the potential drug targets in the tree shrew. Comparisons were then performed between the drug scores of these drug targets and the scores of their human and mouse orthologs (Table S9 in File S1). The top 100 ranked drug targets are shown in Figure 4B. Many of the tree shrew drug targets had higher scores than their mouse orthologs, which were approximately equal to those of their human orthologs (Figure 4B). Among the predicted drug targets in the tree shrew, approximately 57.5% of proteins had higher drug scores than their mouse orthologs. In addition, more than 17% of drug targets in the tree shrew had drug scores comparable to those of their mouse orthologs. These results indicate the similar suitability of the tree shrew and the mouse as drug testing models for depression and various cancers.

### Validation of drug targets

**Figure 5:**
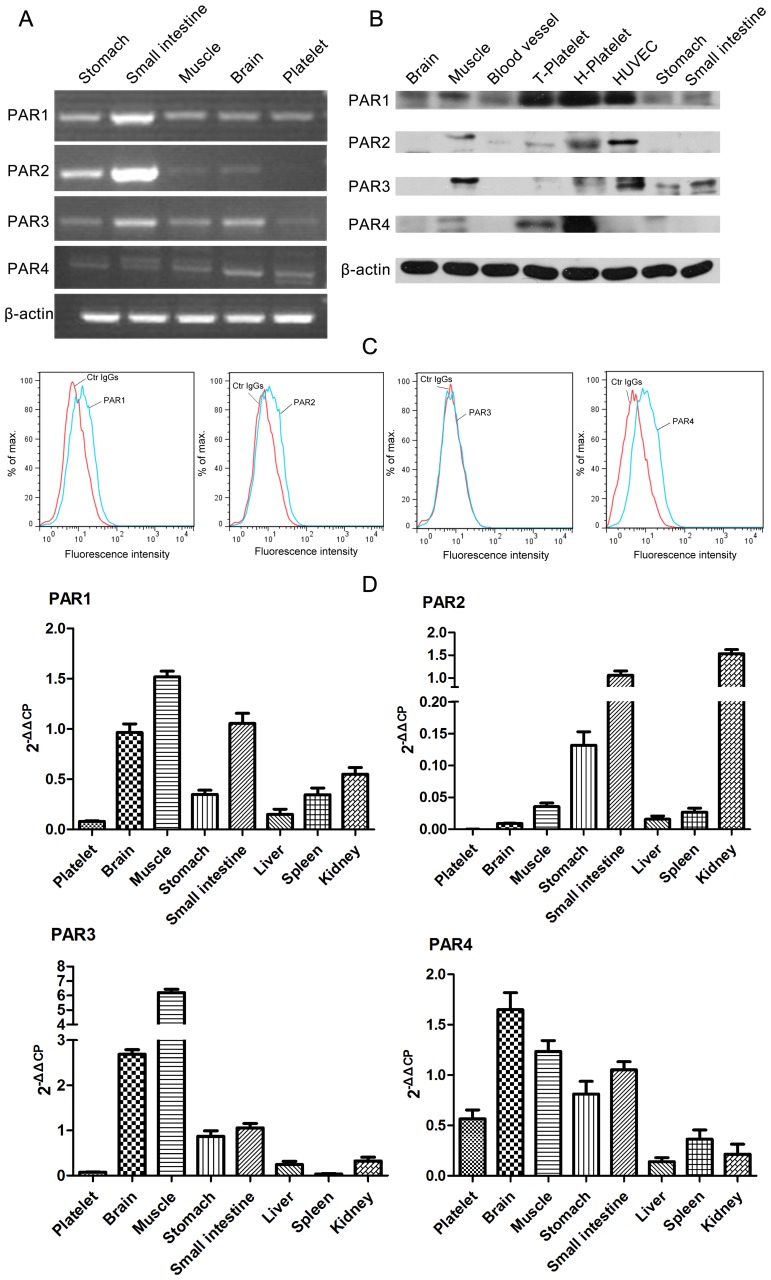
Protease-activated receptors (PARs) present on tree shrew platelets. (A) Amplification of PARs from different tissues using RT-PCR. Primers were designed based on the original tree shrew sequences; β-actin was used as a control. (B) Detection of PAR expression in different tissues by western blotting. PAR anti-human antibodies were used. (C) Surface expression of PAR1 and PAR4 in tree shrew platelets analyzed by flow cytometry. (D) Tree shrew PAR expression measured by qRT-PCR and flow cytometry. The qRT-PCR results are presented as the mean ± SEM (three technical replicates) using β-actin as an internal control.

To illustrate the usefulness and potential of our large-scale approach, we validated the activation of a predicted target using proteinase-activated receptors (PARs), which are members of a GPCR family. The roles of these receptors in cancer as well as in cardiovascular, musculoskeletal, gastrointestinal, respiratory and central nervous system disorders have recently been discovered. PAR expression was detected in the tree shrew, in tree shrews platelets, PAR1 and PAR4 are exclusively detected (Figures 5A and 5D). Similar results were obtained by western blotting (Figure 5B) and flow cytometry analysis (Figure 5C). Furthermore, PAR1, 2, 3 and 4 were found to be highly expressed in tree shrew brain, muscle, stomach and small intestine tissues, at least at the mRNA levels (Figure 5D). These results indicate that the constitutive expression of PAR is similar in tree shrew and human platelets [14] and distinct from that observed in mouse platelets, which express PAR3 and PAR4 [15].

**Figure 6:**
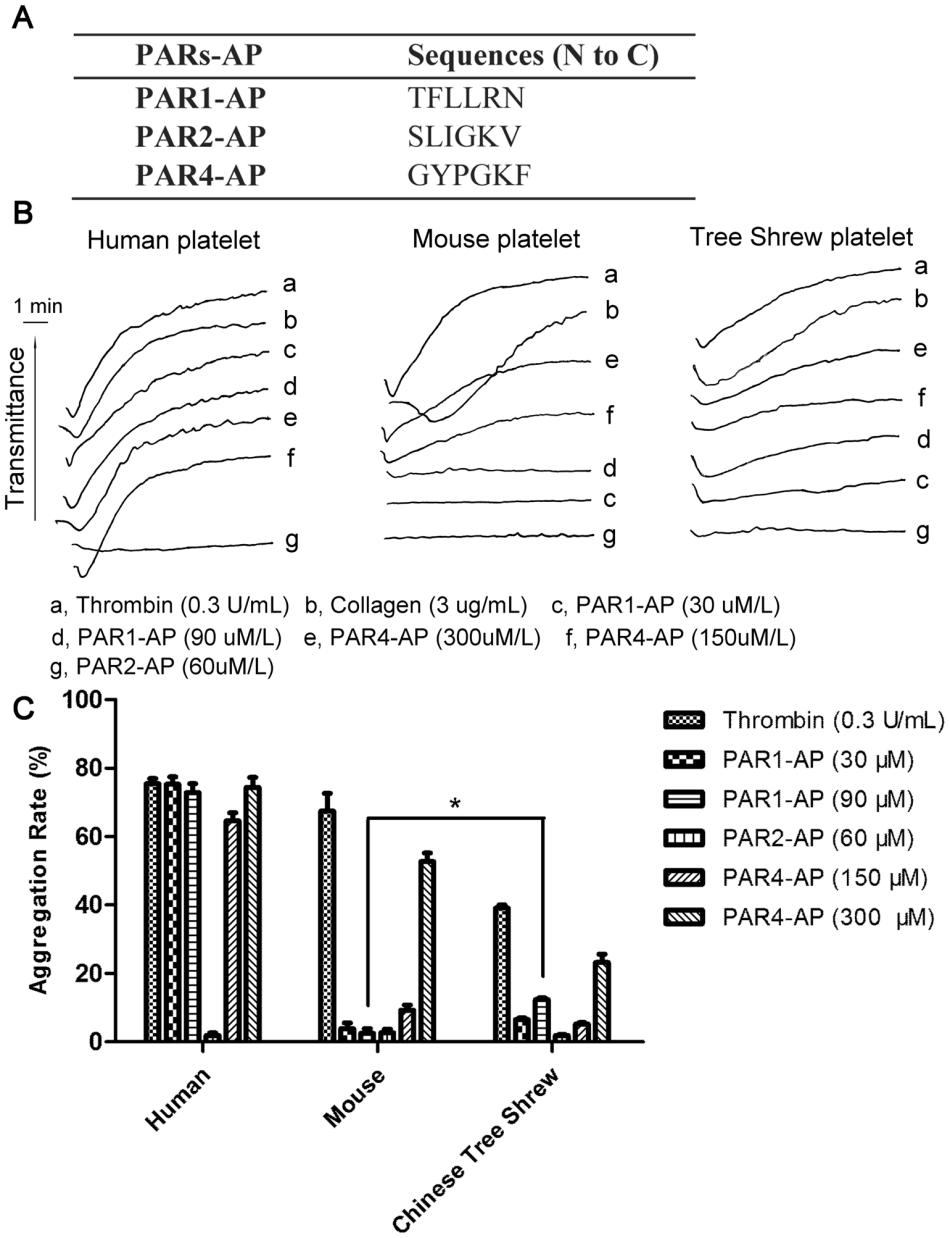
Tree shrew platelets are activated and aggregated by human PAR1-AP and PAR4-AP, whereas mouse platelets are only activated by Human PAR4-AP. (A) Activated peptide (AP) sequences of human PARs with the PAR extracellular N-terminal tethered ligand for the platelet activation assay. (B) Human, mouse and Chinese tree shrew platelets treated with human thrombin (0.3 U/mL), collagen (3 µg/mL), PAR1-AP (30 µM, 90 µM) and PAR4-AP (150 µM, 300 µM). (C) Histogram of the platelet aggregation in the presence of human thrombin, collagen and various human PAR-APs; the “maximum aggregation rate” of each agonist is shown. The results are presented as the mean ± SEM (three technical replicates).

**Figure 7:**
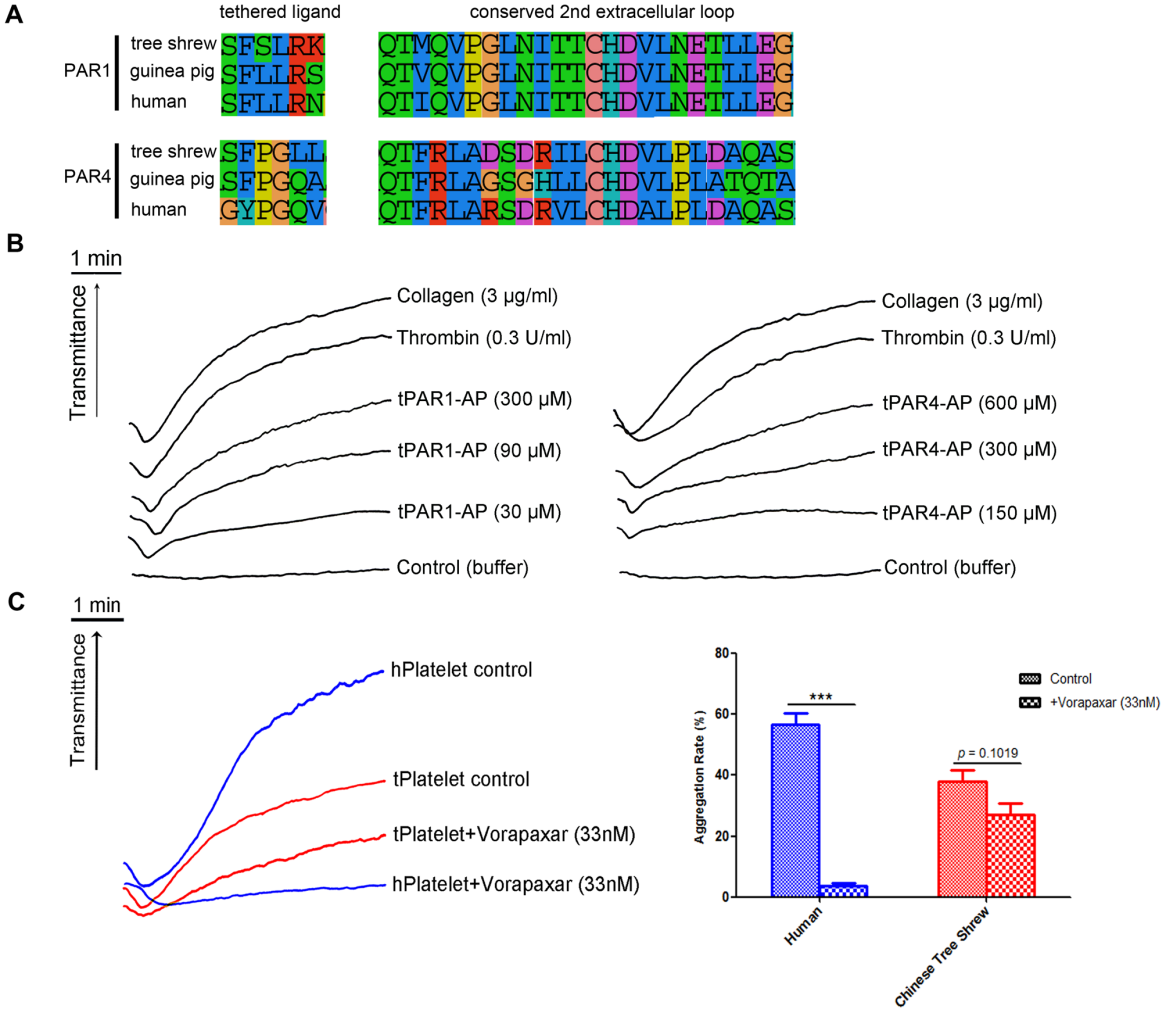
PAR1, 4 of Chinese tree shrews for antithrombotic testing *in vitro*. (A) Protein sequences alignment of PAR1 and 4 from humans, guinea pig and the tree shrew. The data focused on the “tethered ligand” and “conserved 2nd extracellular loop” (ligand binding region) of PAR1 and 4. (B) PAR1 and 4 of the tree shrew can be greatly activated and aggregated by using various concentrations of PAR1-AP (tPAR1-AP: 30 µM, 90 µM, 300 µM ) or PAR4-AP (tPAR4-AP: 150 µM, 300 µM, 600 µM) . Platelet treated with 3 µg/ml collagen and 0.3 U/ml thrombin as positive control; platelet treated with 1 µl buffer B as negative control. (C) Vorapaxar inhibited thrombin-induced human or tree shrew platelet aggregation. Thrombin-induced human platelet aggregation was significantly inhibited with vorapaxar (*p* < 0.001), and only a slight inhibition (*p*  =  0.1019) in the tree shrew. Platelet treated with 0.08U thrombin as the control.

In humans, PARs (PAR1 and PAR4) are responsible for thrombin-induced platelet activation. Platelet activation and aggregation assays were therefore performed to elicit this function using human PAR activated peptides (PAR-APs; Figure 6A). As shown in Figure 6B, tree shrew platelets could be activated by human PAR1-AP and PAR4-AP, whereas mouse platelets were activated only by human PAR4-AP. In particular, when 30 µM PAR1-AP was added, human platelets displayed robust activation with a steep aggregation curve; in mouse and tree shrew platelets, there was no obvious activation and the curve was smooth. When 90 µM PAR1-AP was added, tree shrew platelets exhibited slight activation and a gradually rising curve, whereas no activation was observed in mouse platelets. Both mouse and tree shrew platelets were activated with the addition of PAR4-AP at 150 and 300 µM (Figure 6B). This surprising difference between the tree shrew and mouse platelet response can be attributed to the extracellular N-terminal end of PAR4 that acts as a tethered ligand. Moreover, a significant difference between mouse and Chinese tree shrew was observed on the histogram maked according to the max aggregation rate of human, mouse and Chinese tree shrew platelets (Figure 6C). The aggregation rate of mouse platelets stimulated with PAR1-AP(90 µM) was significant decreased (79.43%) compared to Chinese tree shrew platelets stimulated with PAR1-AP(90 µM). Meanwhile, using the synthesized PAR-APs of Chinese tree shrews, according to alignment results of “tethered ligand region” of PAR1 and PAR4 from humans and Chinese tree shrews (Figure 7A), we observed Chinese tree shrews' platelets had a greater aggregation with tPAR1-AP (90 µM) or tPAR4-AP (300 µM) than stimulated with human PAR1-AP or PAR4-AP (Figure 7B). Under 0.08U thrombin-induced human platelet aggregation assay, a very significant inhibition (*p* < 0.01) was observed after platelets pretreated with 33 nM human PAR1 antagonist vorapaxar for 5 min at 37°C, while in Chinese tree shrews a little weak inhibition ( *p*  =  0.1019 ) was observed (Figure 7C ). These findings shown PAR1 was expressed on the platelet of Chinese tree shrew and was similar with humans, though some differences was also existed between human and Chinese tree shrew PAR1, especially the tethered ligand region and extracellular loop II ligand binding region of PAR1 likely led to the aggregation diversities, some drug testing in platelet could be drawn on the the tree shrews, but not on mice.

## Discussion

Over the last several years, it has become increasingly clear that there are significant differences between rodents, dogs, monkeys and humans with respect to the absorption, distribution and excretion of many drugs [16]. The choice of a suitable animal model for drug testing is thus critical for drug discovery. Because of its genetic characteristics, the tree shrew is a promising biomedical model and ideal for drug testing and drug target analysis. Previous drug target and network analyses have frequently focused on four common drug target families: enzymes, GPCRs, ion channel proteins and nuclear receptors. In addition, immune pathway components are increasingly popular key drug targets for disease therapy [9]. It is well established that high-throughput genome screening can identify potent drug targets. Our large-scale approach, as described in the Results section, was based on the screening of genomic and transcriptomic data of seven candidate drug target families. Based on the ranking of total scores used in our drug target identification approach, more than 90% of the candidate proteins were finally identified as potential drug targets in the tree shrew. Combined with genomic and transcriptomic data, the expression of each drug target is an important criterion during drug evaluation and pharmacological testing. In previous studies, expression profiling of drug targets has been used to compare characteristic cell responses to genetic and chemical perturbations and analyze associations between genes and drugs [17]. The results of our drug target expression analysis reconfirmed the correlation between expression patterns and the results of pharmacological testing and evaluation.

Our drug target analysis identified additional drug targets in the tree shrew that may warrant further drug testing and functional research. The highest-ranked targets were mainly related to cancer, mental disorders and metabolic diseases. Notably, the association with mental disorders may be relevant to the considerable progress that has been achieved in studies of social stress and depression using the tree shrew as a model [7]. In addition, the most impressive drug targets, which were related to various cancers, had the highest scores and most significant expression profiles. Finally, comparison with mouse and human orthologs revealed that more than half of tree shrew drug targets were more comparable to human target proteins than were those in mice. And, most of human drug target proteins are present in the Chinese tree shrew genome. These results encourage us to continue our development of the tree shrew for drug discovery as a potential drug testing model.

To test the applicability of our approach, predicted drug targets of PARs, i.e., subsets of GPCRs, were experimentally validated in the tree shrew. PARs have become attractive targets for the development of novel therapeutics for several diseases [18]. In the treatment of cardiovascular diseases using antiplatelet therapies, PARs are also important targets that bind inhibitors of thrombin-induced platelet activation [19]. Based on our previous studies of PARs and their mediation of platelet aggregation [20], tree shrew PARs were promising targets for platelet aggregation. Importantly, PAR distributions in tree shrew platelets are similar to those of humans but differ from those of mice. In addition, the N-terminal tethered ligand of human PAR-APs can induce the aggregation of tree shrew platelets; in contrast, this tethered ligand does not induce the activation of any mouse PARs except for PAR4. Furthermore, synthesized tree shrew PAR1-AP and PAR4-AP was used to further study the PARs expression on Chinese tree shrews' platelet, at last we found that there was a greater activation than platelets stimulated with human PAR1-AP or PAR4-AP. On the other hand, vorapaxar—an irreversible antagonist of human PAR1, was used in the anti thrombin-induced platelet aggregation assays, our findings shown that vorapaxar had a inhibitory effect on thrombin-induced human or tree shrew platelet aggregation, but human platelets was more sensitive to vorapaxar compared to tree shrew platelets. This result is likely from the difference in PAR1 key site between human and tree shrew, or other unclear factors, such as platelet osmotic pressure. These results collectively validate our prediction that the tree shrew is likely used as a potential research model for drug testing for cardiovascular and other related diseases.

Our approach may be successfully used to predict target proteins for drug testing in animal models. In contrast to network discovery strategies [11], [21], [22], the majority of candidate proteins in our study were predicted as drug targets with a high similarity to human orthologs for further pharmacological testing. If a protein is a new drug target, however, insufficient information exists for further prediction. Nevertheless, our approach is an efficient strategy for large-scale screening of drug targets using genomic or transcriptomic data. In this study, we demonstrated the feasibility of our strategy for large-scale drug target prediction and the evaluation of model-based target identification for drug testing. The Chinese tree shrew is likely useful resource of novel targets for future pharmacological research.

## Materials and Methods

### Ethics statement

All animal studies were reviewed and approved by the Animal Care and Use Committee of the Kunming Institute of Zoology of the Chinese Academy of Sciences.

### Data resources

Chinese tree shrew genomic data were downloaded from the NCBI Nucleotide database (KB320374.1–KB371123.1). RNA-seq data and expression profile data from different tissues were obtained from NCBI SRA (SRA055685) and NCBI GEO (GSE39150).

### Animals

Adult male Chinese tree shrews (*Tupaia belangeri chinensis*) weighting 130−160g were obtained from a breeding colony at the Animal House Center of the Kunming Institute of Zoology, Chinese Academy of Science (Yunnan, China). Adult ICR mice (18–26g) were provided by the Animal Center of Kunming Medical University (Yunnan, China). All animals were provided ad libitum access to food and water. They were housed individually in thermo-regulated rooms (T: 25∼27 °C, RH: 55%∼70%) under a 12 h light/dark cycle (light, 8:00–20:00; dark, 20:00−8:00). After anesthesia , whole blood of Chinese tree shrews and mice were collected from the carotid artery, anti-coagulated with acid-citrate-dextrose.

### Transcriptome assembly

Transcriptome *ab initio* assembly was performed using Tophat [23] and Cufflinks [24] programs with the default parameters. Transcripts from all seven tissues were merged for differential analysis using Cuffdiff. The reference tree shrew genome was downloaded from NCBI as described above. Transcriptome *de novo* assembly was performed with the short-read assembly program SOAP*denovo*-Trans (http://soap.genomics.org.cn/) with the default parameters. The program combined reads with certain lengths of overlap and connected paired-end reads to form contigs. Further assembly of contigs from each sample was performed by sequence splicing and redundancy removal using the sequence clustering software TGICL [25], resulting in the generation of the longest possible non-redundant unigenes. TGICL parameters were identical to those used in our previous study [26].

### Candidate protein identification

Kinase domains were identified in the tree shrew transcriptome using Pfam annotations. Domains were aligned with MAFFT [27], and a phylogenetic tree was constructed using PhyML. Clustering of kinase domains was performed with OrthoMCL [28] using the KINBASE (http://kinase.com/) database as a reference, and the classification results were combined with those of the phylogenetic analysis. HMMTOP [29] was used to identify transmembrane (TM) domains with a filter of ≥ 3 predicted TM domains. After combining these results with those obtained by searching against GPCRDB [30], ranked hits were filtered by homolog-based searching against the NCBI Nr database. GPCR families were also classified using the aforementioned method with OrthoMCL. Ion channel proteins in the tree shrew transcriptome were used as BLAST queries against the LGIC database from the EBI database. The resulting hits were ranked based on *E*-values, and redundant sequences were removed. Neuropeptides were identified using a homolog search against the NeuroPedia database [31]. The best hits were ranked based on *E*-values (1e-10 cutoff). Pre-propeptides were examined for the presence or absence of signal peptide sequences using SignalP 4.0 [32] and mature peptides were then produced. Immune-related genes were identified using a database query search against the tree shrew transcriptome and the Immunome database [33] followed by manual inspection and removal of redundant sequences. Putative proteases and inhibitors were detected by local searching of the MEROPS database [34]. All transcripts with CDSs were batch-submitted for local database searching with the default parameters. Protease and inhibitor gene families were classified based on the hits that were returned. To identify nuclear receptors, we mapped all protein-coding sequences to the KEGG online database and inspected the results manually.

### Weighted profile method

Candidate proteins, predicted as described above, were used as input for our search strategy pipeline. Our method was adapted from that of Tsai [10] with two important modifications: the implementation of a set of default weighting criteria based on search results and the use of weighted profiling. We used four default criteria. The first criterion involved expression in seven tissues (heart, brain, liver, kidney, ovary, testis and pancreas). Candidate proteins represented by FPKM (fragments per kilobase of transcript per million mapped reads) values ≥ 10 were assigned a score of 0.1 from each tissues. The second criterion, the essentiality of genes, indicated that the protein was homologous to mouse orthologs of knockout or lethal phenotypes (mouse phenotype data were retrieved from the Mouse Genome Informatics database). Third, a lead compound score was assigned if a candidate protein had hits in the ChEMBL, DrugBank or TTD (Therapeutic Target Database) drug target databases. Finally, we included a score for GPCR or kinase domains. For weighted profiling, we considered a more generalized method based on the above default criteria. We predicted the total score, *S_total_*(*i*), to have the following interaction profile:
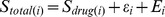
where *ε_i_* is the target essentiality score, *S_drug_*_(*i*)_ is the summed score of the lead compound and *E_i_* is the expression profile of the seven tissues.

We defined the drug weighting profile as
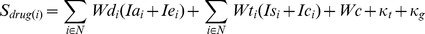
where the variable *N* represents the number of candidate drug targets; *Wd_i_* is the weight of the hits to DrugBank targets; *Ia_i_* and *Ie_i_* represent the number of target hits to approved and experimental drugs, respectively; *Wt_i_* is the weight of the hits to TTD targets; *Is_i_* and *Ic_i_* represent the number of target hits to successful drugs and compounds, respectively; *Wc* is the weight of the hits to ChEMBL targets; and *κ_t_* and *κ_g_* indicate that the drug is considered tractable or druggable, respectively, in ChEMBL.

### Detection of PAR expression

Platelets were isolated at room temperature from a Chinese tree shrew using a modification of the mouse platelet isolation method [35]. In brief, whole blood was collected from the heart, anti-coagulated with acid-citrate-dextrose (ACD), and diluted (1∶1) with Tyrode buffer A (137mM NaCl, 5.6mM glucose, 0.35% BSA, 1 mM MgCl_2_, 2.7 mM KCl, 12 mM NaHCO_3_, 3.3 mM Na_2_HPO_4_, 2.5 mM EDTA, pH 6.5). Platelet-rich plasma (PRP) was isolated by centrifugation at 200g for 20 minutes. Platelets were collected from PRP by centrifugation at 600g for 5 minutes, washed twice with Tyrode buffer A, then resuspended with Tyrode buffer B (137mM NaCl, 5.6mM glucose, 1 mM MgCl_2_, 2.7 mM KCl, 12 mM NaHCO_3_, 3.3 mM Na_2_HPO_4_, pH 7.4). Washed platelets can used for the aggregation assay or inhibit-aggregation assay. Isolated platelet purity was assessed by flow cytometry. We used cDNA as a template for RT-PCR amplification of PARs with specific PAR primers designed based on their transcript sequences. qRT-PCR was performed using a LightCycler 480 system (Roche, Switzerland) with SYBR premix Ex Taq II (Takara, Japan). Samples were run in triplicate. Primers used for qRT-PCR are presented in Table S10 in File S1. For each primer, a melting curve of the PCR product was also generated to ensure the absence of artifacts. The comparative crossing point (*ΔΔ*CP) method [36] was used for the relative quantification of transcript expression. Expression values were normalized using β-actin as an internal control. Whole proteins were extracted from each tissue using protein lysis buffer (50 mM Tris-HCl, 150 mM NaCl and 1% NP-40, pH 7.5) with complete protease inhibitor (Roche, Switzerland). Protein quantification was performed with a protein assay kit (BioRad, USA). The following antibodies (products of Santa Cruz Biotechnology Inc., USA) were used for western blotting: goat anti-human PAR1 antibody C-18, mouse anti-human PAR2 antibody SAM11, mouse anti-human PAR3 antibody 8E8, goat anti-human PAR4 antibody C-20 and mouse anti-human β-actin antibody 5A7 (Abmart, Shanghai, China). PAR expression was detected by flow cytometry (FACS Vantage SE; Becton Dickinson, Franklin Lakes, NJ, USA) using the anti-PAR antibodies used for western blotting with FITC-labeled rabbit anti-goat IgGs and PE-labeled goat anti-mouse IgGs. FITC- and PE-labeled isotype-matched antibodies were used as controls.

### Platelet activation and aggregation assays

Platelets activation and aggregation assays were performed using a platelet aggregometer (Precil, Beijing, China). Human PAR-activated peptides (PAR-APs PAR1-AP, PAR2-AP and PAR4-AP) and Chinese Tree Shrew PAR-APs (tPAR1-AP:SFSLRK, tPAR4-AP:SFPGLL) with a 99% purity were synthesized by GL Biochem (Shanghai, China). Human PAR1 antagonist Vorapaxar (SCH 530348) with a 99.2% purity was purchased from Axon Medchem (Groningen, Netherlands). Washed platelets were treated with PAR-APs, bovine thrombin and collagen (positive control). For the assays of inhibit-platelet aggregation, washed human or Chinese tree shrews' platelets were pre-incubated with Vorapaxar at 37°C for 5 min, then stimulated with thrombin (0.08 U) and the light transmittance were recorded for 5 min with the platelet aggregometer.

### Statistical analysis

The data were representative of three to four independent experiments, and were analyzed with ANOVA. The experimental values are expressed as means ± standard deviations. The level of statistical significance was set at *P* < 0.05.

## Supporting Information

File S1 **Contains additional tables.**
(ZIP)
