# Supplementary material for: Drug Target Mining and Analysis of the Chinese Tree Shrew for Pharmacological Testing
Source: PLoS One. 2014 Aug 8;9(8):e104191. doi: 10.1371/journal.pone.0104191 (PMC4126716; doi:10.1371/journal.pone.0104191)
Supplement: File S1 — Contains additional tables. (ZIP) [file pone.0104191.s001.zip › Supplementary Table 10.doc]

**Table S10. Primers used for PCR and qRT-PCR analysis of tissue specific expression of protease activated receptors (PARs) and designed by Primer-BLAST.**

| **Gene name** | **5’-3’ sense primer** | **5’-3’ antisense primer** |
| --- | --- | --- |
| PAR1 | GAGTCAAAAGCATCCAATGCTACAGT | GAGAGGAAGGCTTACTAAACATACAC |
| PAR2 | GACCTCCTCTCTGTCATCTGGT | GCTTCATGACATACAAAGGGATGGT |
| PAR3 | CAGACATCACCACCTGCCATG | GCAAATGGTAAAAATCACAAGGAT |
| PAR4 | GAGACCCCCAGTATCTACGA | GTGGCCAGCACCCACAGTGCCA |
| β-actin | GAGGCACTCTTCCAGCCATCT | GTGCCAGGGCGGTTATCTCC |
